# Complete Genome Sequence of Rubrobacter xylanophilus Strain AA3-22, Isolated from Arima Onsen in Japan

**DOI:** 10.1128/MRA.00818-19

**Published:** 2019-08-22

**Authors:** Natsuki Tomariguchi, Kentaro Miyazaki

**Affiliations:** a Department of Life Science and Biotechnology, Bioproduction Research Institute, National Institute of Advanced Industrial Science and Technology (AIST), Tsukuba, Ibaraki, Japan; b Faculty of Life Sciences, Toyo University, Itakura, Gunma, Japan; c Department of Computational Biology and Medical Sciences, Graduate School of Frontier Sciences, The University of Tokyo, Kashiwa, Chiba, Japan; Georgia Institute of Technology

## Abstract

Rubrobacter xylanophilus strain AA3-22, belonging to the phylum *Actinobacteria*, was isolated from nonvolcanic Arima Onsen (hot spring) in Japan. Here, we report the complete genome sequence of this organism, which was obtained by combining Oxford Nanopore long-read and Illumina short-read sequencing data.

## ANNOUNCEMENT

The Rubrobacter genus belongs to the *Actinobacteria* and is characterized by radiotolerance. Since the discovery of Rubrobacter radiotolerans (initially named Arthrobacter radiotolerans [1]), several *Rubrobacter* species have been isolated from high-temperature environments (2–6). The organism has gained much attention as a model organism for examining the mechanism for radiotolerance (7–9). To date, six genomic sequences (three complete and three draft genomes, as of 31 July 2019) have been determined (10, 11).

We isolated Rubrobacter xylanophilus from nonvolcanic, oceanic Arima Onsen (hot spring) in Kobe, Japan (34°417′53″N, 135°14′51″E) (12). The environmental sample was spread over Marine agar (Difco) plates and incubated at 65°C. Pink colonies that appeared on the plates after incubation for two nights were examined by 16S rRNA gene sequencing to identify *R. xylanophilus* (99.3% identity to *R. xylanophilus* DSM 9941 16S rRNA gene). The strain, named AA3-22, grew optimally at 58°C, with a doubling time of 13 h.

For genomic DNA extraction, the strain was inoculated in Marine broth (Difco) and cultured at 60°C until saturation. Genomic DNA was prepared using the MagAttract high-molecular-weight (HMW) DNA kit (Qiagen), according to the manufacturer’s instructions (for Gram-positive bacteria).

For long-read sequencing, short genomic fragments were removed using the Short Read Eliminator (Circulomics). Sequencing was performed using a GridION X5 system (Oxford Nanopore Technologies [ONT]); 1.0 μg unfragmented genomic DNA was used for library construction using a ligation sequencing kit (ONT). The prepared library was applied to a FLO-MIN106 R9.41 flow cell (ONT). The long-read sequences, which were base called using Guppy 3.0.3, generated 75,430 reads (639 Mb), with an average length of 8,478.7 bases during a 12-h runtime (data represent reads obtained after quality filtering for average phred quality values above 8.0 using NanoFilt v.2.3.0 [13]). The longest read had 270,703 bases. Default parameters were used for all software.

For short-read sequencing, genomic DNA was sheared to 300-bp fragments by sonication using a Covaris sonicator. The resulting DNA fragments were processed for adaptor ligation and amplified to generate DNA libraries with the Nextera DNA library preparation kit (Illumina). Prepared libraries were subjected to paired-end 2 × 151-base sequencing on the Illumina NovaSeq 6000 platform with sequencing by synthesis (SBS) chemistry. A total of 45,978,934 reads were produced (total read bases, 6.9 Gb). Adapters and low-quality sequencing data were trimmed using fastp v.0.19.5 (14).

For complete *de novo* genome assembly, both long-read and short-read data were processed using Unicycler v.0.4.4 (15), followed by a final polishing step using Pilon v.1.23 (16), generating a single circular contig for the chromosome with a length of 3,022,875 bp (G+C content, 68.8%). Automatic annotation was then performed using the annotation pipeline DFAST v.1.1.0 (17), provided by the DDBJ, which predicted 3,070 coding sequences as well as three rRNA genes and 49 tRNA genes. A JSpecies search (18) in the NCBI genome database revealed that the genomic sequence of AA3-22 showed the highest average nucleotide identity (86.7%) with that of *R. xylanophilus* DSM 9941 (GenBank accession number CP000386, 3,225,748 bp), indicating the species identity of the strain AA3-22 (95% cutoff for the definition of a species [19]). This report will allow for comparative genomic sequence analysis among *Rubrobacter* species that will help deepen our understanding on the radiotolerance of these bacteria.

### Data availability.

This project has been deposited in DDBJ/EMBL/GenBank under the BioProject accession number PRJDB8457. The complete genome sequence of *R. xylanophilus* AA3-22 is available at DDBJ/EMBL/GenBank under the accession number AP019791. Raw sequencing data were deposited in the DDBJ SRA database under the accession number DRA008612 (BioSample number SAMD00177107).
